# Surrogate Data Preserving All the Properties of Ordinal Patterns up to a Certain Length

**DOI:** 10.3390/e21070713

**Published:** 2019-07-22

**Authors:** Yoshito Hirata, Masanori Shiro, José M. Amigó

**Affiliations:** 1 Mathematics and Informatics Center, The University of Tokyo, 7-3-1 Hongo, Bunkyo-ku, Tokyo 113-8656, Japan; 2 Faculty of Engineering, Information and Systems, University of Tsukuba, 1-1-1 Tennodai, Tsukuba, Ibaraki 305-8573, Japan; 3 Human Informatics Research Institute, National Institute of Advanced Industrial Science and Technology, Ibaraki 305-8568, Japan; 4 Centro de Investigación Operativa, Universidad Miguel Hernández, Avda. de la Universidad s/n, 03202 Elche, Spain

**Keywords:** time series analysis, determinism, stochasticity, permutations, hypothesis testing

## Abstract

We propose a method for generating surrogate data that preserves all the properties of ordinal patterns up to a certain length, such as the numbers of allowed/forbidden ordinal patterns and transition likelihoods from ordinal patterns into others. The null hypothesis is that the details of the underlying dynamics do not matter beyond the refinements of ordinal patterns finer than a predefined length. The proposed surrogate data help construct a test of determinism that is free from the common linearity assumption for a null-hypothesis.

## 1. Introduction

Judging whether the underlying dynamics are deterministic or stochastic based on a given time series is an old problem and the first step for modelling such a time series. The current standard approach uses iterative amplitude adjusted Fourier transform (IAAFT) surrogates [1] with some statistics characterizing determinism such as prediction errors [2] and Wayland statistic [3]—but by following this approach, we cannot distinguish nonlinear stochasticity from linear stochasticity or nonlinear determinism.

Recently, we have proposed an alternative approach where we prepare two independent tests for linearity-nonlinearity as well as determinism-stochasticity [4]. For the test of linearity-nonlinearity, we use truncated Fourier transform surrogates (TFTS) [5], an extension of IAAFT surrogates with the mean of $s{\left(t\right)}^{2}s{\left(t+1\right)}^{2}$ over a time series s(t) as a test statistic which is not directly related to the determinism that may exist. For the test of determinism-stochasticity, we use the properties of permutations [6,7,8], which are inequality relations among consecutive measurements: If the underlying dynamics is deterministic and verifies some assumptions (see Section 3 for details), then the number of appearing permutations increases exponentially when the length of permutations is prolonged. But, currently this approach has a problem—we need a long time series of length 1,000,000 to classify stationary time series appropriately [4].

Thus, we propose another approach for testing determinism-stochasticity for the underlying dynamics using the permutation properties of a time series. In this paper, we generate surrogate data which preserve the series of permutations for a given time series almost perfectly and thus the stochastic properties for the underlying dynamics fully up to a certain pre-defined length of the permutations. We call the surrogate data we propose here as entropy preserving surrogates (EPS). Thus, based on the proposed method, we will be able to identify the determinism for the underlying dynamics based on its time series more firmly than by using the existing methods in the literature, helping researchers to make their mathematical model with more confidence.

For the test of linearity-nonlinearity, we continue using TFTS with the mean of $s{\left(t\right)}^{2}s{\left(t+1\right)}^{2}$ as the statistic. If (s(t),s(t+1)) follows the multivariate Gaussian distribution, a higher-order moment such as $s{\left(t\right)}^{2}s{\left(t+1\right)}^{2}$ can be characterized with the means and variances [9] and becomes pivotal [10] and constant if the underlying dynamics are kept. Thus, any variation can be attributed to a deviation from the linear Gaussianity. Hence, the mean of $s{\left(t\right)}^{2}s{\left(t+1\right)}^{2}$ can be used as a test statistic for nonlinearity. We demonstrate the proposed set of methods using time series of length 1000.

## 2. Our Mathematical Settings

Before starting the main parts of this manuscript, we define our mathematical settings more rigorously.

Our interest is on a dynamical system f:X×P→X on a manifold *X* driven by a parameter space *P*, which may change along the time. Thus, typically, we have

(1) x(t+1)=f(x(t),p(t))

for x(t)∈X and p(t)∈P, starting from the initial conditions x(0)∈X and p(0)∈P. We cannot directly observe x(t). Instead, we have an observation function g:X→R such that s(t)=g(x(t)). When *g* is given by a skew product of the state *X* and its disturbance *Q*, then we can model observational noise as well.

Then, our question is whether p(t) is constant throughout the time or p(t) changes along the time. If p(t) is constant throughout the time, then we call the underlying dynamics deterministic. If p(t) changes along the time in a deterministic way, then we also call the underlying dynamics deterministic. If p(t) changes along the time randomly, then we call the underlying dynamics stochastic.

## 3. Background

There have been a number of researches in the existing literature discussing how to characterize determinism and/or stochasticity: The best known approaches could be the ones using the parallelness of neighboring orbits [3,11] and the optimal neighborhood size for local linear predictions [12]. Recently, the most popular one could be that by Amigó et al. [8,13,14], which uses the fact that there exist forbidden ordinal patterns. To explain the approach of Amigó et al. (2008) [8] in more detail, we first define ordinal patterns or permutations [6].

Suppose that a time series is given by s(t)∈R. We focus on inequality relations among consecutive measurements s(t),s(t+1),…,s(t+L−1) over time period between *t* and t+L−1. Namely, if we order these measurements in the ascending order, we could have $s\left(t+i_{1}\right)\leq s\left(t+i_{2}\right)\leq \cdots \leq s\left(t+i_{L}\right)$, where $i_{j}\in \left\{0,1,2,\ldots ,L-1\right\}$ and are unique. For convenience, we define s(t+i)≤s(t+j) if s(t+i)=s(t+j) and i<j. Then, the corresponding permutation is $\pi \left(\left\{s\right\},t,L\right)=\left(i_{1},i_{2},\ldots ,i_{L}\right)$.

The number of appearing permutations increases exponentially when the length of permutations *L* is prolonged if the underlying dynamics is one-dimensional, deterministic and piecewise monotone [13] or, in any dimension, if the underlying dynamics is deterministic and expansive [7].

Thus, Amigó et al. (2008) [8] uses the existence of forbidden permutations as the signature for a deterministic system. The contraposition of this theorem was previously used for identifying if a given time series is generated from a nonlinear and stochastic system [4].

Moreover, the entropies obtained using the permutation statistics can be used for estimating the metric and topological entropies [6,7,15].

Therefore, permutations are good tools for characterizing time series generated from the underlying dynamics.

## 4. Methods

**Figure 1:**
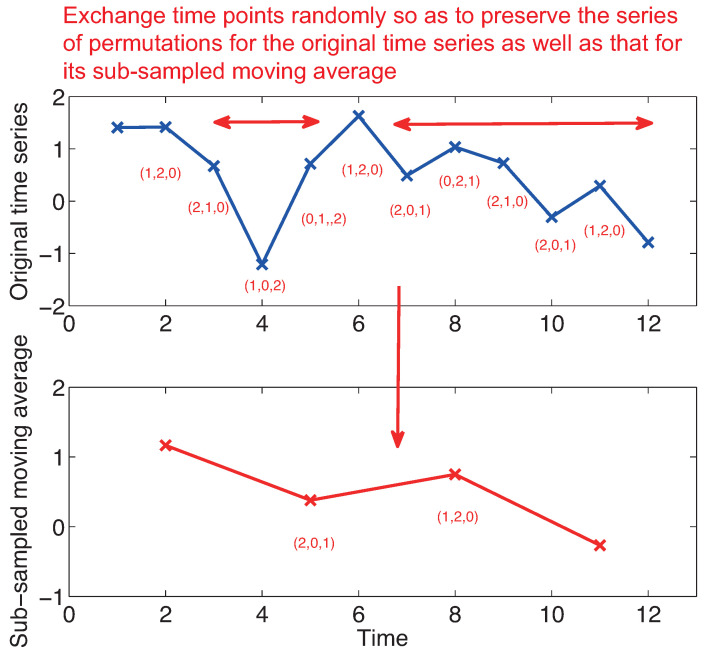
Schematic figure showing how we generate an entropy preserving surrogate.

Here we propose to generate surrogate data that preserves all the statistical properties of permutations up to a certain predefined length *L*. Here we call such surrogate data as entropy preserving surrogates (EPS). Our method is quite simple and follows a general principle proposed by Schreiber (1998) [16]: we randomly exchange the temporal order of time series through the method of simulated annealing [17] so that we preserve a series of permutations for a given time series as well as a series of permutations for a moving average of the given time series over length *L* subsampled by an interval *L* (see Figure 1). In this way, we generate 39 surrogate data for obtaining the significance level of 2/(39+1)=5% level for each time series. Since a series of permutations is preserved in the entropy preserving surrogates, all the transitions from every permutation to another are preserved. Therefore, our null hypothesis in the entropy preserving surrogates is that the underlying dynamics has significant historical dependence only up to the length *L* and the dependence over *L* does not matter for the underlying dynamics. As a by-product, permutation entropies calculated up to length *L* are preserved. Please find the detail on how to generate EPS in the Appendix A.

To compare an original time series with its surrogate data for telling whether the original time series is statistically different from its surrogate data or not, we estimate the maximal Lyapunov exponent in the following way: First, we fit the parameters a(t) and b(t) for the following local linear model for each time *t* using 20 neighboring points in infinite-dimensional delay coordinates [18]:

(2) s(t+1)−s(τ+1)≈a(t)+b(t)(s(t)−s(τ)),

where s(τ) is one of 20 spatial neighbors for s(t). Then, we evaluate the following quantity as a test statistic for the second half of each dataset:

(3) $$E_{t}\left[\log|b\left(t\right)|\right].$$

This statistic can be regarded as a proxy for the maximal Lyapunov exponent. We decided to use 20 neighbors for the above estimation because if the number of neighbors is less than 20, then the estimation would heavily depend on the closer neighbors, while the estimation would not be able to characterize local states well if the number of neighbors is greater than 20.

## 5. Results

### 5.1. Toy Examples

First, we show some numerical experiments for datasets generated from toy models which are free from observational noise. We set L=30 throughout the paper because we would like to investigate the deterministic structure which finely persists over the pseudo-periodicity evaluated by the pseudo-periodic surrogates [19]. To obtain pseudo-periodic surrogates, we used the three-dimensional delay coordinates with delay 8.

Our first toy model is the first-order autoregressive linear (AR(1)) model [20]. The model we used is as follows:

(4) x(t+1)=0.8x(t)+η(t),

where η(t) follows the Gaussian distribution of mean 0 and standard deviation 1.

The second toy model is the GARCH model [21]. The model equations are

(5) y(t)=0.409933+0.095y(t−1)+ϵ(t),

(6) $$h\left(t\right)=14.4038+0.095\epsilon {\left(t-1\right)}^{2}+0.895h\left(t-1\right),$$

where ϵ(t) follows the Gaussian distribution of mean 0 and standard deviation $\sqrt{h(t)}$. We observe y(t) to generate a time series.

The third toy model is the model for noise-induced order [22]. We use the following equations:

(7) $$x\left(t+1\right)=f\left(x\left(t\right)\right)+b+10^{-2.5}u\left(t\right),$$

(8) $$f\left(x\right)=\left\{\begin{array}{cc} \left(-{\left(0.125-x\right)}^{1/3}+0.50607357\right)\exp\left(-x\right), & \mathrm{if}\;x<0.125,\\ \left({\left(x-0.125\right)}^{1/3}+0.50607357\right)\exp\left(-x\right), & \mathrm{if}\;0.125\leq x<0.3,\\ 0.121205602\left({\left(10x\exp\left(-10\frac{x}{3}\right)\right)}^{19}\right), & \mathrm{otherwise}, \end{array}\right.$$

where u(t) follows the uniform distribution between −1 and 1.

The fourth toy model is the logistic map [23]. We use the following equation:

(9) x(t+1)=3.8x(t)(1−x(t)).

We also use time-continuous models for testing the proposed method. Our fifth toy model is the Lorenz model [24]. We use the following equations:

(10) $$\dot{x}=-10\left(x-y\right),$$

(11) $$\dot{y}=-xz+28x-y,$$

(12) $$\dot{z}=xy-\frac{8}{3}z.$$

We sampled *x* every 0.1 unit time.

The sixth model is the Rössler model [25]. Here we use the following equations:

(13) $$\dot{x}=-\left(y+z\right),$$

(14) $$\dot{y}=x+0.36y,$$

(15) $$\dot{z}=0.4+z\left(x-4.5\right).$$

We sampled *x* every 1 unit time.

For each model, we generated 20 time series of length 1000 to examine the robustness for the proposed test. In this paper, we also used pseudo-periodic surrogates [19] with correlation dimensions [26] as test statistics. For pseudo-periodic surrogates, the null hypothesis is that the underlying dynamics has determinism beyond pseudo-periodicity. Such surrogate data can be generated by connecting segments of time series by choosing a neighboring point at each step with a Gaussian uncertainty. If we generate surrogate data in this way, a rough periodicity related to the underlying dynamics is preserved, while fine structure is destroyed. Thus, we can judge if there is determinism beyond this rough periodicity. For the cases without observation noise, we also use the proxy for the maximal Lyapunov exponent as a test statistic for pseudo-periodic surrogates for comparison.

When we use TFTS, we apply the end-to-end matching [27] using the first and last 20 points to suppress the artificial high-frequency components which might be generated during applying the Fourier transforms. When we generate the proposed entropy preserving surrogates, we use the same segments of time series, which could be the reason why we can find slight differences between the values for the proxies of the maximal Lyapunov exponent between Figures 6 and 7 as we will discuss later.

In all the model analyses down below, we used whole the datasets for each time series, meaning that we did not divide each time series into halves or so.

**Figure 2:**
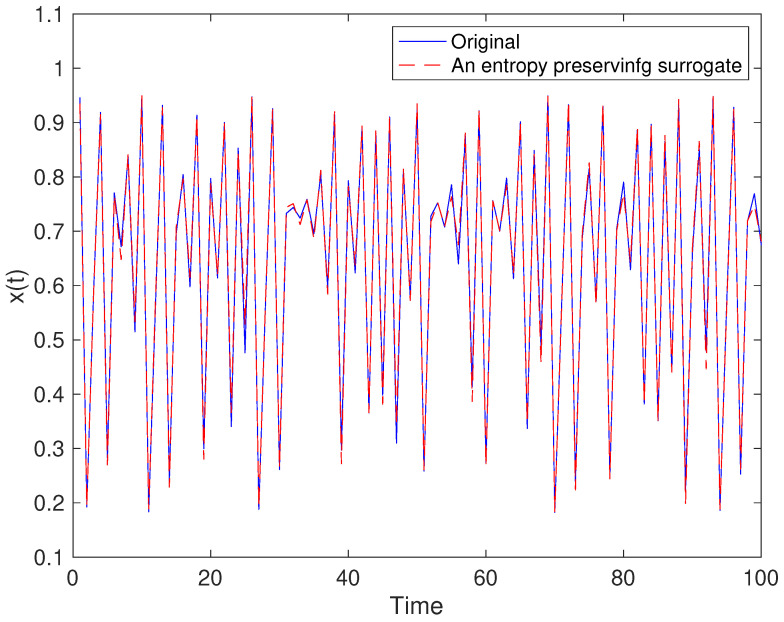
Example of an entropy preserving surrogate for the logistic map.

**Figure 3:**
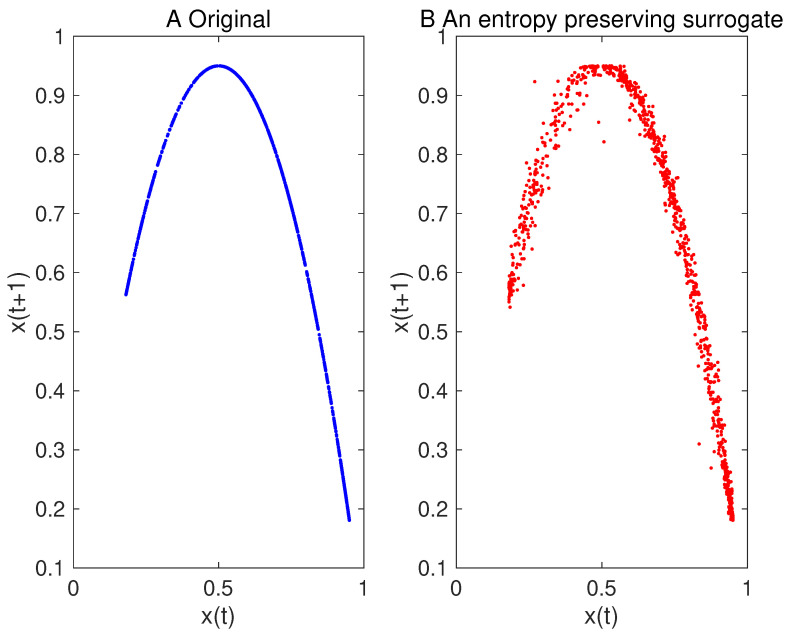
Return plot for the original time series of the logistic map (**A**) and that for one of its entropy preserving surrogates (**B**).

Examples for entropy preserving surrogates are shown in Figure 2 and Figure 3. Especially, such a time series shown for entropy preserving surrogates looks similar to the original time series (Figure 2). When we look at their return plots, we can see that an entropy preserving surrogate (Figure 3B) seems to be perturbed from the original time series (Figure 3A).

**Figure 4:**
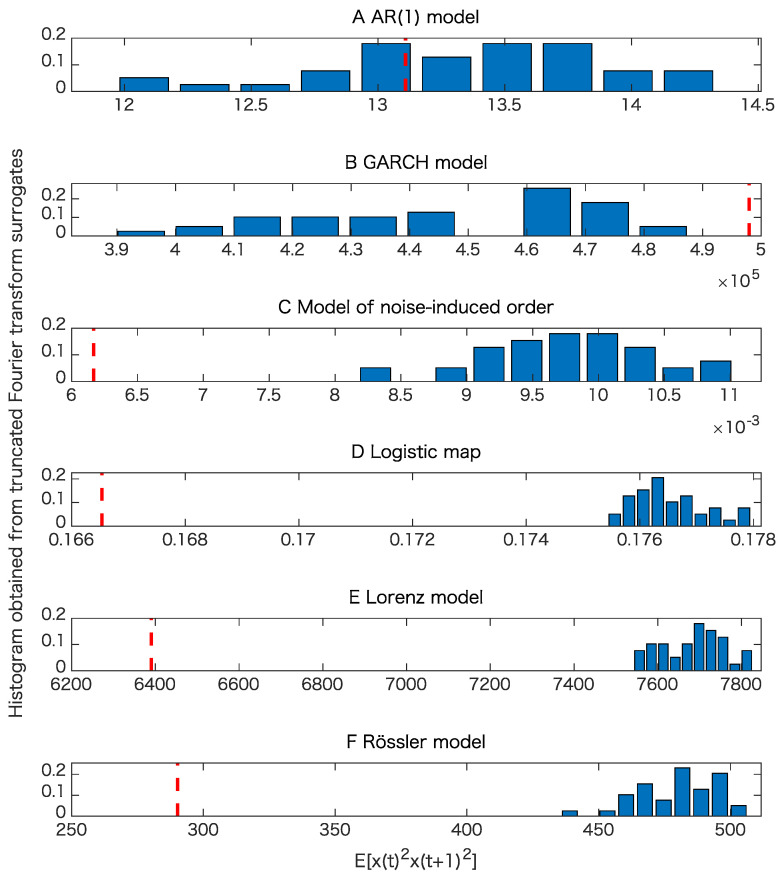
Examples of tests for nonlinearity for various models when the datasets are free from observational noise. Here an important point is whether or not the value obtained from each original time series shown in the red vertical dashed line is within the interval specified with the minimum and the maximum for the test statistic $E[x{\left(t\right)}^{2}x{\left(t+1\right)}^{2}]$ of the 39 truncated Fourier transform surrogates (TFTS) surrogates, which can be interpreted from each histogram. Therefore, it does not matter much whether the test statistic obtained from the original data is smaller or greater than those obtained from TFTS surrogates. (**A**) result for the AR(1) model; (**B**) result for the GARCH model; (**C**) result for the model of noise-induced order; (**D**) result for the logistic map; (**E**) result for the Lorenz model; (**F**) result for the Rössler model.

**Figure 5:**
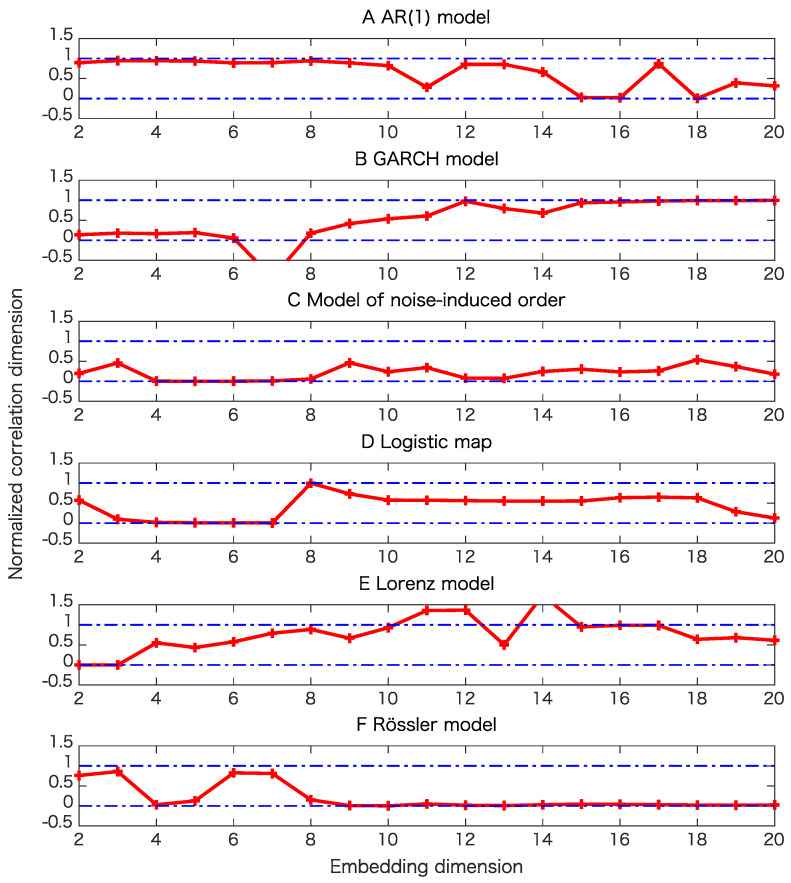
Examples of tests of determinism beyond pseudo-periodicity using pseudo-periodic surrogates for various models when the datasets are free from observational noise. Here we use the correlation dimensions as test statistics. In this surrogate data, rough periodic behavior is preserved, while fine structure related to the possible underlying determinism in question is destroyed. Correlation dimensions are normalized so that the minimum and the maximum values for the correlation dimensions of the 39 pseudo-periodic surrogates for each dimension become 0 and 1, respectively. (**A**) result for the AR(1) model; (**B**) result for the GARCH model; (**C**) result for the model of noise-induced order; (**D**) result for the logistic map; (**E**) result for the Lorenz model; (**F**) result for the Rössler model.

**Figure 6:**
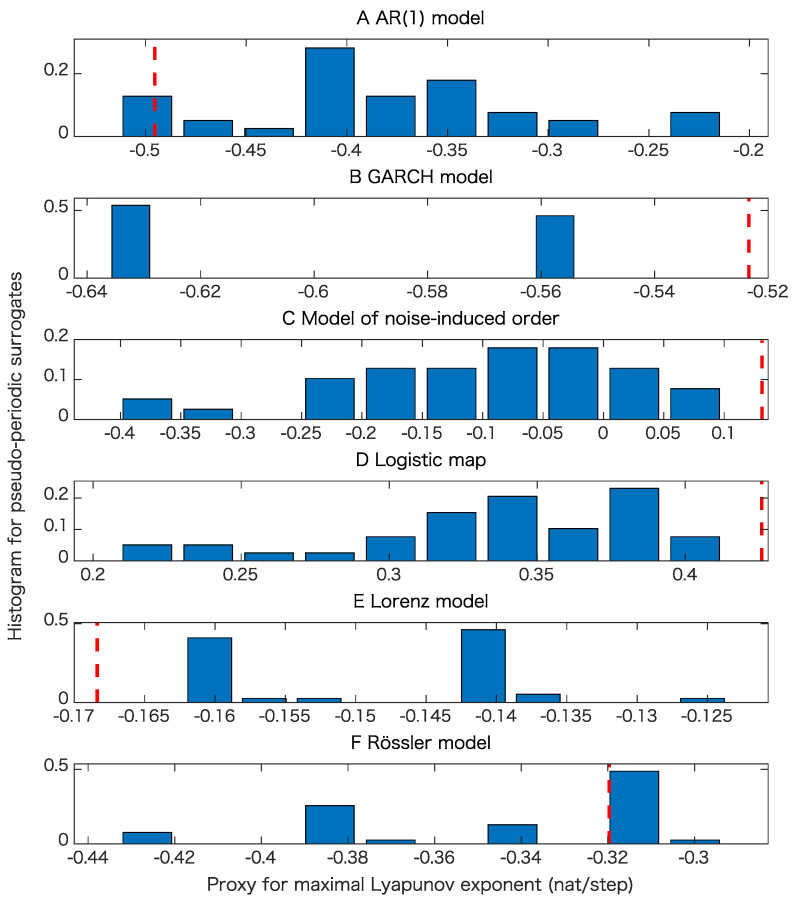
Examples of tests of determinism beyond pseudo-periodicity using pseudo-periodic surrogates when we use the proxy for the maximal Lyapunov exponent as a test statistic. In each panel, the red dashed line corresponds to the value obtained from the original time series and the histogram, obtained from the pseudo-periodic surrogates. (**A**) result for the AR(1) model; (**B**) result for the GARCH model; (**C**) result for the model of noise-induced order; (**D**) result for the logistic map; (**E**) result for the Lorenz model; (**F**) result for the Rössler model.

**Figure 7:**
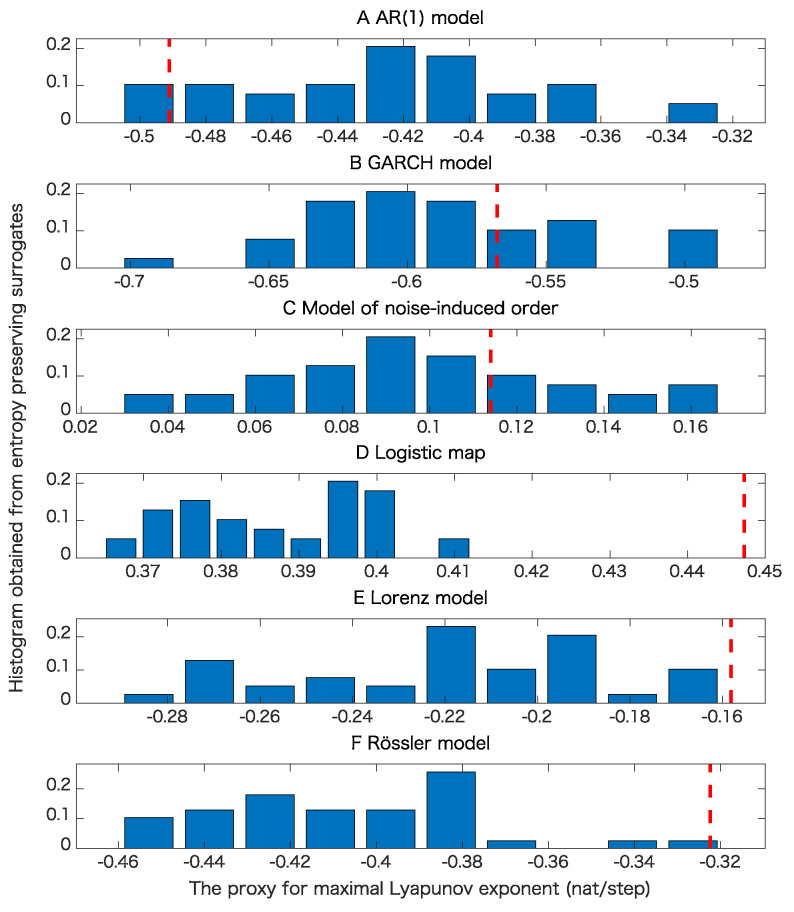
Examples of tests of determinism beyond 30 steps using the proposed entropy preserving surrogates for various models when the datasets are free from observational noise. In each panel, the red dashed line corresponds to the value of test statistic obtained from the original data. (**A**) result for the AR(1) model; (**B**) result for the GARCH model; (**C**) result for the model of noise-induced order; (**D**) result for the logistic map; (**E**) result for the Lorenz model; (**F**) result for the Rössler model.

The results of the surrogate tests are summarized in Figure 4, Figure 5, Figure 6 and Figure 7 as well as Table 1. For most cases, the tested time series were classified into the correct classes for the corresponding toy models. To evaluate the numbers of rejections appropriately, consider the binomial distribution with N=20 trials and p=0.05. Then, the cumulative sum of probabilities from 0 becomes more than 95% if the number of positives is 4 or greater. For example, 3 rejections for the test of linearity for the AR(1) model are not statistically significant. For the same reason, 3 rejections for the proposed test of determinism beyond 30 steps for the model of noise-induced order are not statistically significant.

The results presented in Figure 4 and Table 1 show that the nonlinearity test examined here is robust.

Table 1 and comparison of Figure 5B,C and Figure 6B,C mean that the results of pseudo-periodic surrogates heavily depend on test statistics we use. These results may be due to the fact that pseudo-periodic surrogates are typical realizations and need a pivotal statistic for the test [10].

Figure 7C,D mean that a time series with the same value for the permutation entropies up to length 30 is likely to have a positive Lyapunov exponent even if the underlying dynamics is stochastic. This usage could lead to another implication obtained from the entropy preserving surrogates. The results shown in Table 1 mean that the proposed method has some skill for detecting the determinism for the underlying dynamics.

The results presented in Table 1 also show that the proposed method works well even for flows such as the Lorenz model and Rössler models as far as sampling intervals are chosen appropriately.

**Figure 8:**
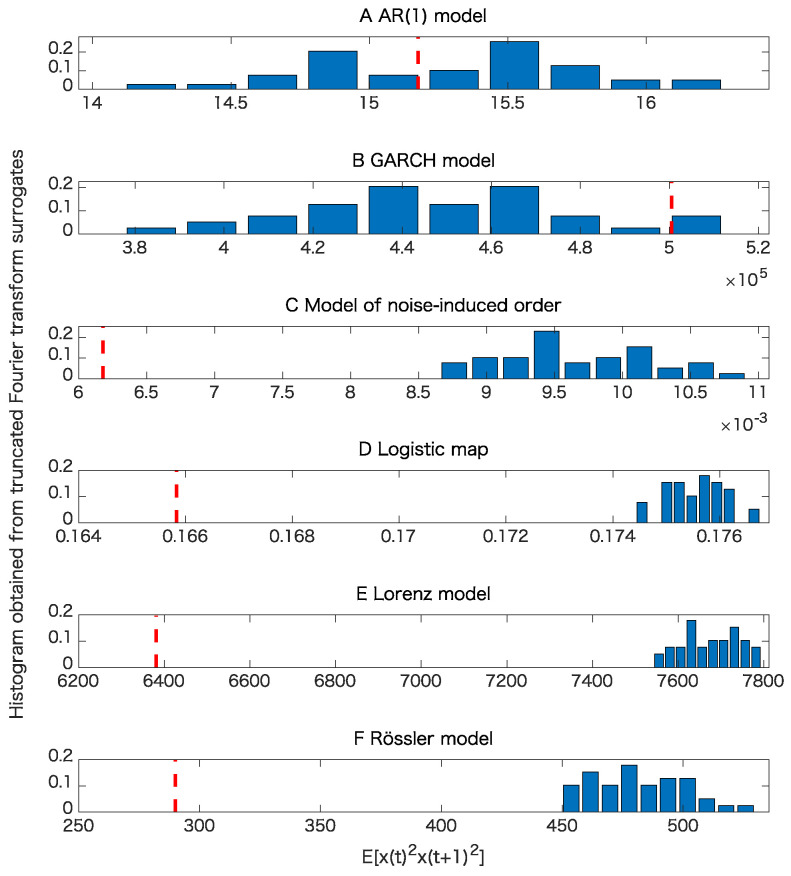
Examples of tests of nonlinearity for various models when 5% observational noise is added. See the caption of Figure 5 to interpret the results. (**A**) result for the AR(1) model; (**B**) result for the GARCH model; (**C**) result for the model of noise-induced order; (**D**) result for the logistic map; (**E**) result for the Lorenz model; (**F**) result for the Rössler model.

**Figure 9:**
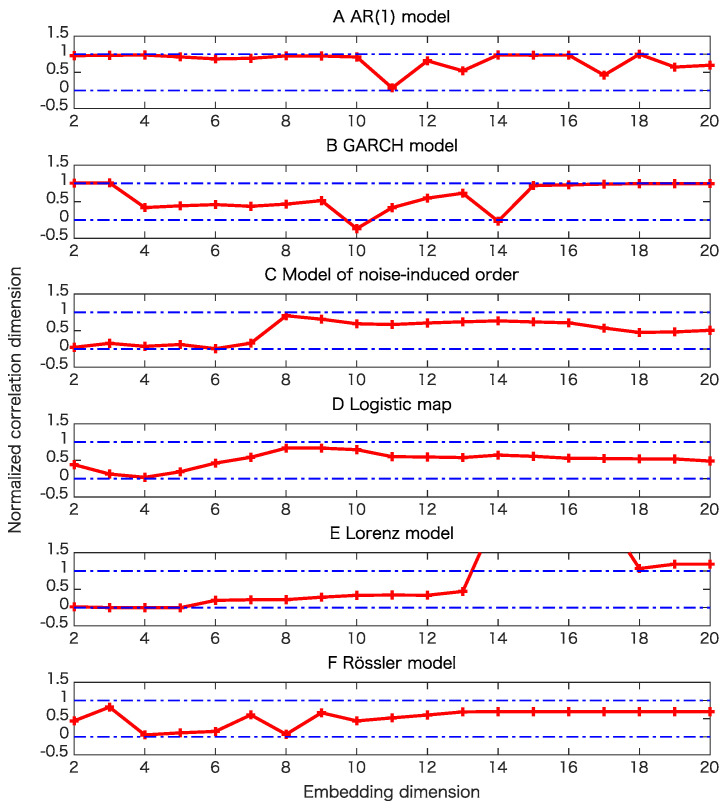
Examples of tests of determinism beyond pseudo-periodicity using pseudo-periodic surrogates for various models when 5% observational noise is added. (**A**) result for the AR(1) model; (**B**) result for the GARCH model; (**C**) result for the model of noise-induced order; (**D**) result for the logistic map; (**E**) result for the Lorenz model; (**F**) result for the Rössler model.

**Figure 10:**
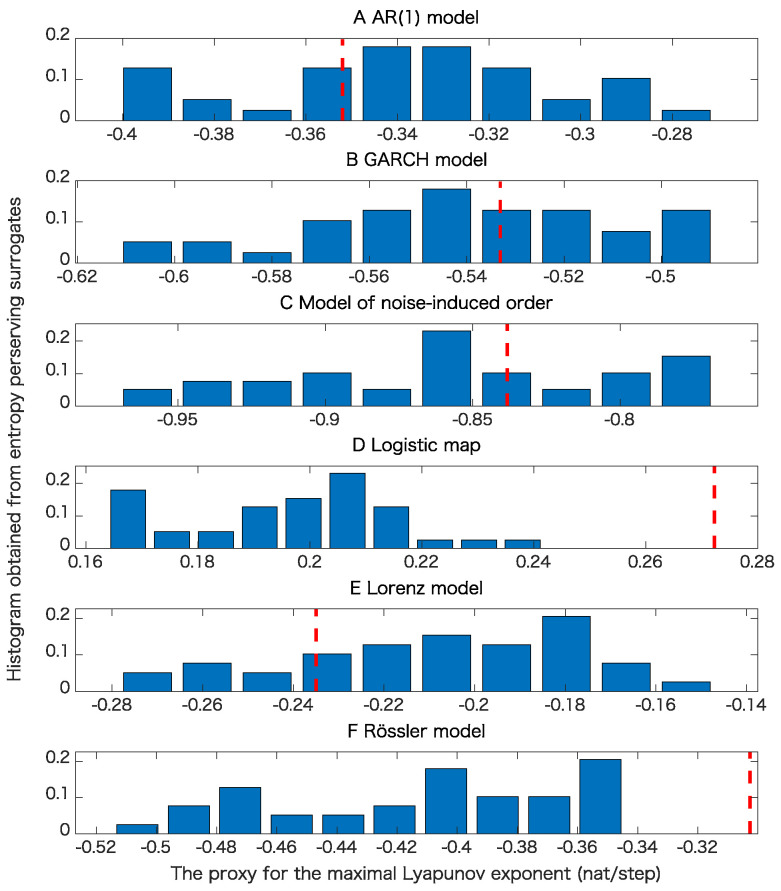
Examples of tests of determinism using the proposed entropy preserving surrogates for various models when 5% observational noise is added. (**A**) result for the AR(1) model; (**B**) result for the GARCH model; (**C**) result for the model of noise-induced order; (**D**) result for the logistic map; (**E**) result for the Lorenz model; (**F**) result for the Rössler model.

We also tested the cases where for each case, we added Gaussian observational noise of mean 0 and standard deviation which is 5% of the standard deviation of the original time series (Figure 8, Figure 9 and Figure 10, and Table 2). But, still the proposed method seems to work properly. Determinism beyond pseudo-periodicity was detected for the GARCH model and the model of noise-induced order, while the determinism was weak in the sense that the dependence did not persist beyond 30 steps statistically significantly. On the other hand, the logistic map tended to exhibit determinism beyond 30 steps (Table 2). Overall, Table 2 shows the robustness for the proposed method against observational noise.

### 5.2. Real Data Example of the USD/JPY Market

We analyzed the dataset of the USD/JPY market compiled by the Thomson Reuters Cooperation. The record starts from 1 January 2006 and ends on 31 December 2015. We use the first 100,000 quotes for the analysis here. We divided the dataset by every 1000 quotes into 100 segments, and took inter-quote intervals for each segment.

**Figure 11:**
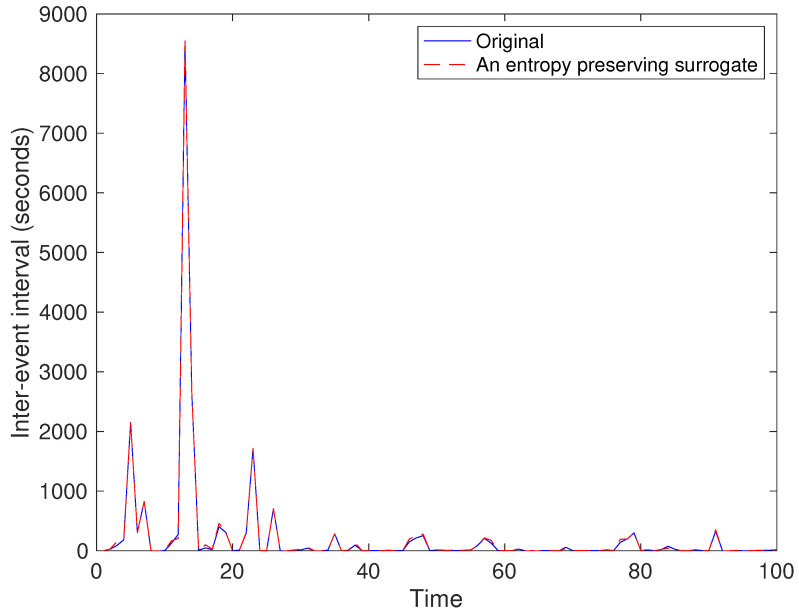
Example of an entropy preserving surrogate for a part of the USD/JPY data.

**Figure 12:**
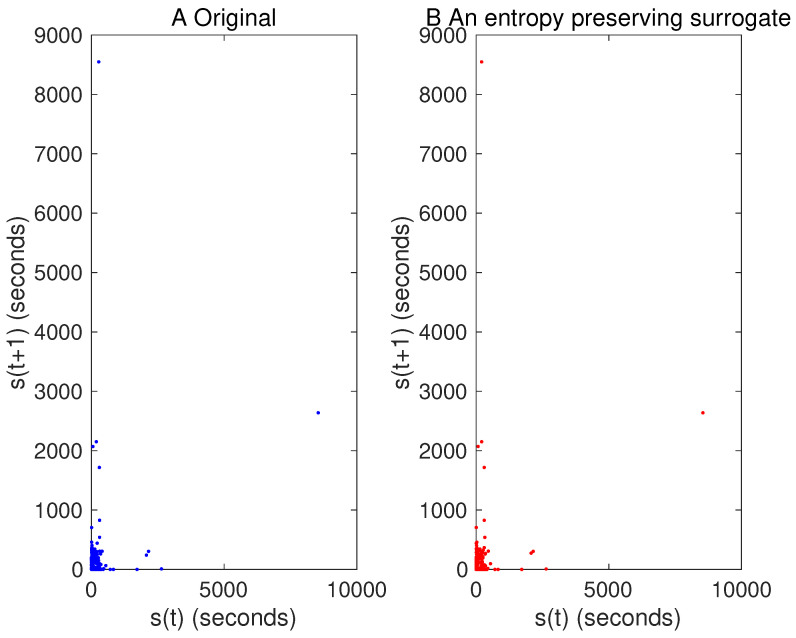
Return plot for the original time series of a USD/JPY data part (**A**) and that for one of its entropy preserving surrogates (**B**).

For the first segment, one of generated entropy preserving surrogates looks as shown in Figure 11 and Figure 12. We can see that typical characteristics for the time series as well as return plots are almost preserved.

The results are summarized as Table 3. Nonlinearity was detected in 24 out of 100 cases, while determinism beyond 30 steps was detected in 12 out of 100 cases. Because these numbers are significant from the viewpoint of the binomial distribution of 100 trials and the probability 0.05 for each test, namely judging from the facts that each test is 5% significant and each time segment is independent from each other, overall, the dataset of the USD/JPY market seems nonlinear with the determinism beyond 30 quotes.

## 6. Discussions

**Figure 13:**
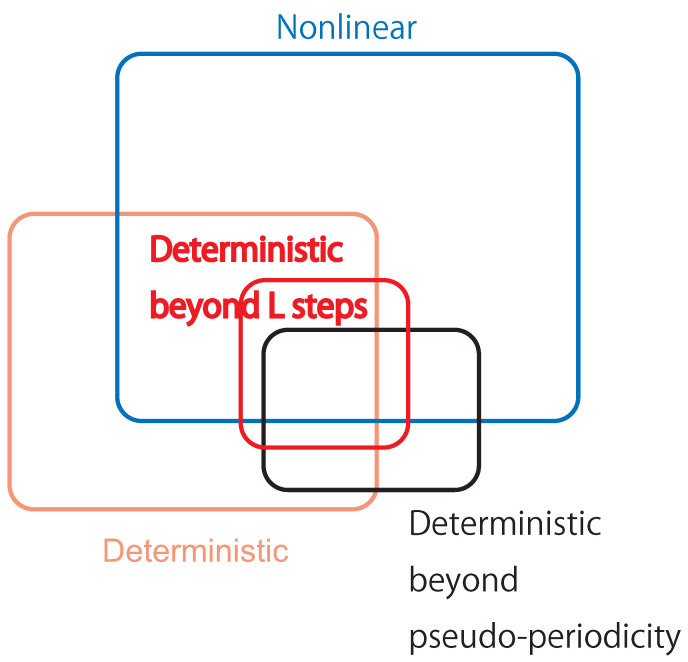
The Venn diagram describing the relationship among original properties for the underlying dynamics such as nonlinearity and determinism against properties we can identify with surrogate data such as determinism beyond pseudo-periodicity (pseudo-periodic surrogates [19]) and determinism beyond *L* steps (the proposed entropy preserving surrogates).

Although we set L=30 in this manuscript, we may vary the length *L* of permutations for elucidating the effect and the length of dynamical dependence. By choosing *L*, we can control the length of dependence which should have significant meaning. Thus, by varying *L*, we can narrow down the topical area of a target time series mostly into the intersection of nonlinear and deterministic regions, whose regions could be smaller than the region specified with pseudo-periodic surrogates as shown in Figure 13. Hence, together with the methods [5,19] in the existing literature, the proposed entropy preserving surrogate helps us to specify the assumptions of a model more finely when we try to construct a model based on a time series.

For pseudo-periodic surrogates, the length 1000 of time series might have been too short to show the determinism beyond pseudo-periodicity for the dataset of the USD/JPY data, while it was sufficient to show the determinism beyond 30 quotes using the proposed method. Thus, we would like to explore the effect for the length of time series in the future more deeply.

The proposed method preserves series of permutations for the original time series as well as that for its sub-sampled moving average. Thus, the proposed method of entropy preserving surrogates can be regarded as a constrained realization [10] rather than a typical realization. When we focus on surrogate data generated by permutations, there are methods such as those of References [28,29,30]. Because these methods are surrogate data as typical realizations, the proposed method is the first method generating surrogate data with permutations as a constrained realization. As a constrained realization, the proposed method can formally be used with a non-pivotal statistic [10], which does not have to provide a consistent value for a class of null models. Thus, we hope that the proposed method be powerful for investigating the deterministic properties beyond a pre-defined length for a given time series.

If there are a pair of time series and we generate entropy preserving surrogates for both, then we can also preserve symbolic transfer entropies [31] and transcripts [32]. Therefore, applying entropy preserving surrogates to multivariate data time series could be an interesting and open problem.

## 7. Conclusions

We have proposed a method for generating surrogate data such that all the properties of permutations up to a certain length are preserved. Such surrogate data look very similar to the original data as shown in Figure 2 and Figure 11, but with dynamical noise especially demonstrated in Figure 3. By using the four toy models, we evaluated that the proposed method works finely. Then, we applied the proposed method to inter-quote interval data in the USD/JPY market and found that the market behaved in a nonlinear and deterministic manner, which is consistent with our previous findings [33].

## Figures and Tables

**Table 1:** Results of noise free data summarized as classifications. We counted the number of rejections for each test for each model. The italic numbers correspond to the significant numbers of rejections based on the calculations using the binomial distributions.

Property∖Model	AR(1)	GARCH	Noise-Induced Order	Logistic	Lorenz	Rössler
Nonlinearity with $E[x{\left(t\right)}^{2}x{\left(t+1\right)}^{2}]$	3	*20*	*20*	*20*	*20*	*20*
Determinism beyond pseudo-periodicity with correlation dimensions	0	*20*	0	0	0	*20*
Determinism beyond psuedo-periodicity with maximal Lyapunov exponent	1	1	*7*	*17*	2	0
Determinism beyond 30 steps with maximal Lyapunov exponent	2	1	3	*20*	*8*	*6*
Total	20	20	20	20	20	20

**Table 2:** Results of 5% observational noise data summarized as classifications. See the caption of Table 1 to interpret the results.

Property∖Model	AR(1)	GARCH	Noise-Induced Order	Logistic	Lorenz	Rössler
Nonlinearity with $E[x{\left(t\right)}^{2}x{\left(t+1\right)}^{2}]$	1	*19*	*20*	*20*	*20*	*20*
Determinism beyond pseudo-periodicity with correlation dimensions	0	*20*	*20*	0	*20*	*11*
Determinism beyond 30 steps with maximal Lyapunov exponent	3	0	2	*16*	*6*	*7*
Total	20	20	20	20	20	20

**Table 3:** Results of the USD/JPY data summarized as classifications. See the caption of Table 1 to interpret the results.

Property	Number of Time Segments
Nonlinearity with $E[x{\left(t\right)}^{2}x{\left(t+1\right)}^{2}]$	*24*
Determinism beyond pseudo-periodicity with correlation dimensions	0
Determinism beyond 30 steps with maximal Lyapunov exponent	*12*
Total	100

## Appendix A

Let {s(t)∈R|t=1,2,…,T} be a given time series. Then, its moving average of *L* consecutive points sub-sampled by every *L* time points can be defined by $\left\{\bar{s}\left(u\right)=\frac{1}{L}\sum_{i=1}^{L}s\left(\left(u-1\right)L+i\right)|u=1,2,\ldots ,\left\lfloor T/L\right\rfloor \right\}$.

First, we convert the given time series {s(t)} and its moving average $\left\{\bar{s}\right\}$ to the corresponding permutation series {π({s},t,L)|t=1,2,…,T−L+1} and $\left\{\pi \left(\left\{\bar{s}\right\},u,L\right)|u=1,2,\ldots ,\left\lfloor T/L\right\rfloor -L+1\right\}$.

Second, we initialize our simulated annealing algorithm by setting the current time series {c(t)} to the original time series {s(t)}.

Third, we repeat the following process until the number of iterations reaches (NS+10)×S, where we set NS=39, which is the number of surrogate data, and *S* = 10,000, which is the number of iterations we skip:

1. Increment the current number *i* of iterations by 1.
2. Prepare an attempt a(t) for replacement by swapping two elements of {c(t)}.
3. Calculate {π({a},t,L)|t=1,2,…,T−L+1} and $\left\{\pi \left(\left\{\bar{a}\right\},u,L\right)|u=1,2,\ldots ,\left\lfloor T/L\right\rfloor -L+1\right\}$.
4. Calculate the number of differences between $\left[\left\{\pi \left(\left\{s\right\},t,L\right)|t=1,2,\ldots ,T-L+1\right\},\left\{\pi \left(\left\{\bar{s}\right\},u,L\right)|u=1,2,\ldots ,\left\lfloor T/L\right\rfloor -L+1\right\}\right]$ and $\left[\left\{\pi \left(\left\{a\right\},t,L\right)|t=1,2,\ldots ,T-L+1\right\},\left\{\pi \left(\left\{\bar{a}\right\},u,L\right)|u=1,2,\ldots ,\left\lfloor T/L\right\rfloor -L+1\right\}\right]$. Let #n denote this number.
5. Let *p* be the probability for accepting the attempt, which can be calculated as exp[−iβ#n].
6. Generate a uniform random number between 0 and 1. If the random number is less than *p*, then replace the current time series {c(t)} by the attempt {a(t)}.
7. If *i* is a multiple of *S* and i>10S, then record the current {c(t)} as the (i/S−10)-th surrogate data.
